# Perinatal outcomes of twin pregnancies with preterm premature rupture of the membranes at 24–34 weeks’ gestation

**DOI:** 10.1038/s41598-021-02884-x

**Published:** 2021-12-03

**Authors:** Shuwei Zhou, Yajun Yang, XiaoYan Zhang, Xiaoling Mu, Quan Quan, Qimei Zhong, Lingwei Mei, Lan Wang

**Affiliations:** 1 Department of Obstetrics, Chongqing Health Center for Women and Children, No. 120 Longshan Road, Chongqing, 401120 China; 2 Department of Gynecology, The First Affiliated Hospital of Chongqing Medical University, No. 1, YouYi Road, Chongqing, 400016 China

**Keywords:** Diseases, Medical research, Risk factors

## Abstract

To describe the perinatal outcomes of twin pregnancies with preterm premature rupture of membranes (PPROM) before 34 weeks’ gestation and identify factors associated with discharge without severe or moderate-severe neonatal morbidity. This study was conducted as a retrospective analysis of twin pregnancies with PPROM occurring at 24 0/7 to 33 6/7 weeks’ gestation. Perinatal outcomes were assessed by gestational age (GA) at PPROM and compared between PPROM and non PPROM twins. Factors associated with discharge without severe or moderate-severe neonatal morbidity were identified using logistic regression analysis. Of the 180 pregnancies (360 foetuses), only 17 (9.4%) women remained pregnant 7 days after PPROM. There were 10 (2.8%) cases of prenatal or neonatal death; 303 (84.2%) and 177 (49.2%) neonates were discharged without severe or moderate-severe morbidity, respectively. As GA at PPROM increased, the adverse obstetric and neonatal outcomes decreased, especially after 32 weeks. There was no significant difference in general neonatal outcomes between PPROM and non PPROM twins. The GA at PPROM and latency period were both significantly associated with discharge without severe or moderate-severe neonatal morbidity. Pregnancy complications and 5-min Apgar score < 7 increased severe neonatal morbidity. As GA at PPROM increased, the risk of adverse perinatal outcomes decreased. GA at PPROM and latency period were significantly associated with discharge without severe or moderate-severe neonatal morbidity.

## Introduction

Preterm premature rupture of membranes (PPROM), defined as membrane rupture before 37 weeks’ gestation, accounts for 3% of all pregnancies^1^. Foetuses experiencing PPROM face increased risks of maternal-foetal infection, cord prolapse, placental abruption, and intrauterine death^2^. Prematurity, which is the primary neonatal consequence of PPROM, is a major contributor to perinatal morbidity and mortality, and 25–30% of preterm births are attributed to PPROM^3^. Consequently, the occurrence of neonatal morbidity, such as respiratory distress syndrome (RDS), sepsis, intraventricular haemorrhage (IVH), necrotising enterocolitis (NEC), bronchopulmonary dysplasia (BPD), retinopathy of prematurity (ROP), and even mortality, is increased after PPROM^4^. Expectant management is recommended for pregnant women with PPROM before 34 0/7 weeks’ gestation if no maternal or foetal contraindications exist^2^.

The rate of twin pregnancies is gradually increasing because of the widespread use of assisted reproductive technology and ovulation induction. PPROM and preterm birth are more common in twin pregnancies: the incidence of PPROM in twin pregnancies is 7–10% versus only 2–4% for singleton pregnancies^5^. Twin pregnancies are more likely to have a shorter duration from membrane rupture to delivery, a higher risk of earlier delivery, and adverse neonatal outcomes than singleton pregnancies^6,7^. Previous studies of twin pregnancies with PPROM compared their clinical characteristics and outcomes with those of singleton pregnancies and had small sample sizes, administered antenatal antibiotics, magnesium sulphate, and corticosteroid variably or included different gestational age (GA) ranges^8–10^. Moreover, the perinatal outcomes of PPROM in twin pregnancies have not been extensively described until now.

The present study aimed to describe the obstetric and neonatal outcomes of twin pregnancies with PPROM at 24 0/7 to 33 6/7 weeks’ gestation stratified by GA at membrane rupture and identify factors associated with discharge without severe or moderate-severe morbidity.

## Results

### Patient selection

**Figure 1:**
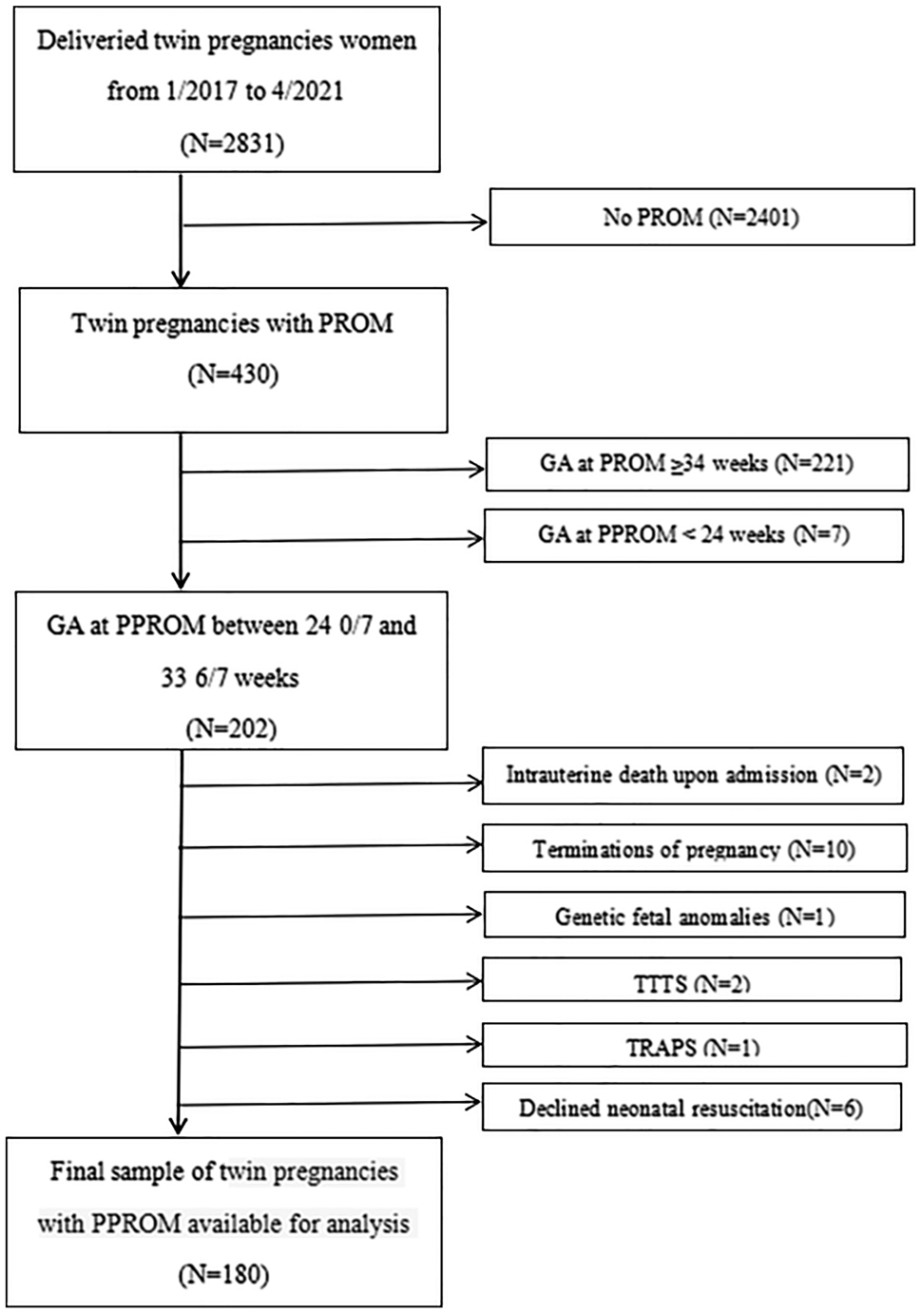
Flow chart of the study population. Legend: *GA* gestational age, *PROM* premature rupture of membranes, *PPROM* preterm premature rupture of membranes.

Of the 2831 women with twin pregnancies who delivered at the Obstetrics Department between January 2017 and April 2021, 430 with premature rupture of membranes were identified; among them, 209 had PPROM at < 34 0/7 weeks’ gestation, while seven had PPROM at < 24 0/7 weeks’ gestation. Of the 202 women with PPROM between 24 0/7 and 33 6/7 weeks’ gestation, 10 were excluded due to termination of pregnancy, two due to intrauterine death upon admission, one due to genetic foetal anomalies, two due to TTTS, one due to TRAPS, and six because the couple had declined neonatal resuscitation. The remaining 180 women and their infants were analysed (Fig. 1).

### Maternal characteristics

The demographic and clinical characteristics of twin pregnancies with PPROM are shown in Table 1. All pregnant women were administered antibiotics upon admission, and almost all of them received antenatal steroids and magnesium sulphate as long as the pregnancy did not require immediate termination; only 99 (55%) patients received complete steroid treatment. Of the 180 patients, eight had undergone a previous caesarean section, while none had a history of preterm births or PPROM in a prior pregnancy. The probability of a pregnant woman having gestational diabetes, ICP, and HDP with any of the three pregnancy complications was 37.8%. The median maternal age was 30 years (Q1:28 to Q3:32 years). The median GA at PPROM was 32 2/7 weeks (Q1:30 1/7 to Q3: 33 1/7 weeks). The median latency period was 20 h, and merely 65 (36.1%) and 17 (9.4%) women in the study cohort remained pregnant 48 h and 7 days after PPROM, respectively.

**Table 1:** Demographic and clinical characteristics of twin pregnancies with PPROM.

Variable	Value
Age (y)	30 (28–32)
Prepregnancy BMI (kg/m2)	21.35 (19.37–23.62)
Nulliparous	164 (91.1)
At work	91 (50.6)
DCDA	158 (87.8)
Conception by ART	137 (76.1)
Complete steroids treatment	99 (55.0)
Use of tocolytic agent	58 (32.2)
Any pregnancy complications	68 (37.8)
Gestational diabetes	49 (27.2)
ICP	19 (10.6)
HDP	14 (7.8)
GA at PPROM	32 2/7 (30 1/7–33 1/7)
24 0/7–27 6/7	15 (8.3)
28 0/7–29 6/7	23 (12.8)
30 0/7–31 6/7	35 (19.4)
32 0/7–33 6/7	107 (59.4)
Median latency period (h)	20 (7–72)
Latency period ≥ 24 h	84 (46.7)
Latency period ≥ 48 h	65 (36.1)
Latency period ≥ 7d	17 (9.4)
Birth weight (Kg)	1.77 (1.49–1.97)
Female fetes	170 (47.2)

Data are median (IQR, Q1-Q3) or *N* (%).

*BMI* body mass index, *DCDA* dichorionic diamniotic, *ART* assisted reproductive technology, *ICP* intrahepatic cholestasis of pregnancy, *HDP* hypertensive disorder of pregnancy, *GA* gestational age, *PPROM* pretem premature rupture of membranes.

### Clinical outcomes

Table 2 depicts the clinical outcomes by GA at PPROM. The median GA at delivery was 32 4/7 weeks (Q1: 31 0/7 to Q3: 33 3/7 weeks). The incidence of chorioamnionitis was 10%, that of placental abruption was 5.6%, that of cord prolapse was 4.4%, and that of caesarean delivery was 91.1% in entire study cohort. When we treated GA at PPROM as a continuous variable, a negative correlation was noted between GA at PPROM and the occurrence of chorioamnionitis (*P* < 0.01; correlation coefficient [CC] =  − 0.227) and cord prolapse (*P* < 0.01, CC =  − 0.116). GA at PPROM was negatively correlated with the latency period (*P* < 0.01, CC =  − 0.217). As GA at PPROM increased, caesarean delivery ((*P* < 0.01, CC = 0.125) and birth weight (*P* < 0.01; CC = 0.568) increased, while the rate of NICU admissions (*P* < 0.01, CC =  − 0.347), length of neonatal hospital stay (*P* < 0.01, CC =  − 0.442), and cost of neonatal hospitalisation (*P* < 0.01, CC =  − 0.492) declined. The longest length of stay in the neonatal ward was 104 days, and the highest hospitalisation cost for newborn care reached 186 thousand CNY. When GA at PPROM was divided into < 32 and ≥ 32 weeks, the above correlation was more obvious. In addition, there was a weak positive correlation between neonatal sepsis and clinical chorioamnionitis (P = 0.037, CC = 0.11).

**Table 2:** Clinical outcomes by GA at membrane rupture for twin pregnancies.

Outcome	GA at PPROM (weeks)			
	24 0/7–27 6/7 (*N* = 15)	28 0/7–29 6/7 (*N* = 23)	30 0/7–31 6/7 (*N* = 35)	32 0/7–33 6/7 (*N* = 107)
Latency period (h)	67 (8–152)	32 (11–153)	64 (7–137)	13 (6–48)
Latency period ≥ 24 h	9 (60.0)	14 (60.9)	21 (60.0)	40 (37.4)
Latency period ≥ 48 h	9 (60.0)	10 (43.5)	19 (54.3)	27 (25.2)
Latency period ≥ 7d	3 (20.0)	4 (17.4)	7 (20.0)	3 (2.8)
GA at delivery (wk)	27 4/7 (26 5/7–28 1/7)	29 5/7 (29 0/7–29 6/7)	31 3/7 (31 0/7–31 6/7)	33 2/7 (32 5/7–33 5/7)
Cesarean delivery	12 (80.0)	20 (87.0)	32 (91.4)	100 (93.5)
Clinical chorioamnionitis	4 (26.7)	5 (21.7)	5 (14.3)	4 (3.7)
Placental abruption	2 (13.3)	2 (8.7)	1 (2.9)	5 (4.7)
Cord Prolapse	2 (13.3)	3 (13.0)	1 (2.9)	2 (1.9)
5-min Apgar score < 7	6 (20.0)	2 (4.3)	0 (0)	4 (1.9)
Admission to NICU	27 (96.4)	32 (69.6)	24 (34.3)	38 (17.8)
Neonatal hospital stay (d)	55 (19–71)	40 (34–46)	21 (15–32)	13 (8–18)
Birth weight (Kg)	1.02 (0.91–1.20)	1.34 (1.22–1.43)	1.59 (1.46–1.73)	1.90 (1.76–2.08)
Neonatal hospitalisation cost(thousand CNY)	77 (53–97)	57 (46–72)	26 (17–38)	15 (10–22)

Data are median (Q1-Q3) or *N* (%).

*GA* gestational age, *NICU* neonatal intensive care unit, *CNY* China Yuan.

### Neonatal outcomes

The neonatal morbidity and mortality per pregnancy and per neonate associated with PPROM in twin pregnancies by GA at membrane rupture are reported in Table 3. Of the 360 foetuses, two from one pregnancy experienced antepartum demise and eight died in the NICU; hence, 350 survived to hospital discharge. GA at PPROM of nine dead foetuses or newborns was 24 0/7 to 27 6/7 weeks; one newborn died of cardiogenic shock after membrane rupture at 33 3/7 weeks. Major morbidities noted in the 358 newborns who survived at birth were PDA in 146 (40.8%); IVH in 28 (7.8%), including two with grade III or IV IVH; ROP in 41 (11.5%) and none with grade 3 or 4 ROP; BPD in two (7.8%); NEC in 41 (11.5%), including 24 with stage IIB or III NEC; RDS in 120 (33.5%); sepsis in 80 (22.3%); respiratory failure in 124 (34.6%); severe pneumonia in 29 (8.1%); need for mechanical ventilation in 185 (51.7%); and none with periventricular leukomalacia during hospitalisation. Except for IVH (*P* = 0.052) and severe pneumonia (*P* = 0.262), as GA at PPROM (treated as a continuous variable) increased, the risk of other adverse neonatal outcomes decreased (all *P* < 0.001). Compared to patients with GA < 32 weeks at PPROM, those with GA ≥ 32 weeks at PPROM had significantly lower neonatal morbidity and mortality rates (*P* < 0.001), including IVH (*P* = 0.021) but excluding severe pneumonia (*P* = 0.188).

**Table 3:** Neonatal morbidity and mortality associated with PPROM in twin pregnancies by GA at membrane rupture.

Variable (weeks’ gestation)	Per pregnancy	Per neonate
**Patent ductus arteriosus**	54.2 (46.8–61.6)	40.8 (35.7–45.9)
24 0/7–27 6/7	92.9 (66.1–99.8)	75.0 (57.9–92.1)
28 0/7–29 6/7	73.9 (54.5–93.3)	56.5 (41.6–71.4)
30 0/7–31 6/7	51.4 (34.0–68.8)	34.3 (22.9–45.7)
32 0/7–33 6/7	45.8 (36.2–55.4)	35.0 (28.6–41.5)
**Intraventricular hemorrhage**	14.0 (8.8–19.1)	7.8 (5.0–10.6)
24 0/7–27 6/7	21.4 (4.7–50.8)	14.3 (4.0–32.7)
28 0/7–29 6/7	21.7 (3.5–40.0)	13.0 (2.9–23.2)
30 0/7–31 6/7	20.0 (6.1–33.9)	10.0 (2.8–17.2)
32 0/7–33 6/7	9.3 (3.7–15.0)	5.1 (2.2–8.1)
**Retinopathy of prematurity**	17.3 (11.7–22.9)	11.5 (8.1–14.8)
24 0/7–27 6/7	64.3 (35.1–87.2)	42.9 (23.3–62.4)
28 0/7–29 6/7	60.9 (39.3–82.4)	45.7 (30.7–60.6)
30 0/7–31 6/7	17.1 (14.0–30.3)	8.6 (1.8–15.3)
32 0/7–33 6/7	1.9 (0.2–6.6)	0.9 (0.1–3.3)
**Bronchopulmonary dysplasia**	11.2 (6.5–15.8)	7.8 (5.0–10.6)
24 0/7–27 6/7	64.3 (35.1–87.2)	50.0 (30.3–69.7)
28 0/7–29 6/7	26.1 (6.7–45.5)	15.2 (14.4–26.0)
30 0/7–31 6/7	11.4 (3.2–26.7)	8.6 (1.8–15.3)
32 0/7–33 6/7	0.9 (0–5.1)	0.5 (0–2.6)
**Necrotising enterocolitis**	20.7 (14.7–26.7)	11.5 (8.1–14.8)
24 0/7–27 6/7	28.2 (8.4–58.1)	17.9 (6.1–36.9)
28 0/7–29 6/7	52.2 (30.1–74.3)	28.3 (14.7–41.8)
30 0/7–31 6/7	25.7 (10.5–40.9)	14.3 (5.9–22.7)
32 0/7–33 6/7	11.2 (5.1–17.3)	6.1 (2.8–9.3)
**Sepsis**	37.4 (30.3–44.6)	22.3 (18.0–26.7)
24 0/7–27 6/7	64.3 (35.1–87.2)	46.4 (26.7–66.1)
28 0/7–29 6/7	78.3 (60.0–96.5)	45.7 (30.7–60.6)
30 0/7–31 6/7	42.9 (25.6–60.1)	25.7 (15.2–36.2)
32 0/7–33 6/7	23.4 (15.2–31.5)	13.1 (8.5–17.6)
**Respiratory failure**	52.0 (44.6–59.3)	34.6 (29.7–39.6)
24 0/7–27 6/7	92.9 (66.1–99.8)	64.3 (45.4–83.2)
28 0/7–29 6/7	91.3 (72.0–98.9)	67.4 (53.3–81.5)
30 0/7–31 6/7	51.4 (34.0–68.8)	34.3 (22.9–45.7)
32 0/7–33 6/7	38.3 (29.0–47.7)	23.8 (17.1–29.6)
**Severe pneumonia**	15.1 (9.8–20.4)	8.1 (5.3–10.9)
24 0/7–27 6/7	21.4 (4.7–50.8)	10.7 (2.3–28.2)
28 0/7–29 6/7	13.0 (2.8–33.6)	6.5 (1.4–17.9)
30 0/7–31 6/7	22.9 (8.2–37.5)	12.9 (4.8–20.9)
32 0/7–33 6/7	12.1 (5.9–18.4)	6.5 (3.2–9.9)
**Respiratory distress syndrome**	42.5 (35.1–49.8)	33.5 (28.6–38.4)
24 0/7–27 6/7	92.9 (66.1–99.8)	89.3 (71.8–97.7)
28 0/7–29 6/7	100 (85.2–100)	93.5 (82.1–98.6)
30 0/7–31 6/7	45.7 (28.4–63.1)	34.3 (22.9–45.7)
32 0/7–33 6/7	22.4 (14.4–30.5)	13.1 (8.5–17.6)
**Need for mechanical ventilation**	64.8 (57.7–71.9)	51.7 (46.5–56.9)
24 0/7–27 6/7	100 (76.8–100)	96.4 (81.7–99.9)
28 0/7–29 6/7	100 (85.2–100)	95.7 (85.2–99.5)
30 0/7–31 6/7	71.4 (55.7–87.2)	57.1 (45.3–69.0)
32 0/7–33 6/7	50.5 (40.8–60.1)	34.6 (28.2–41.0)
**Neonatal or foetal death**	4.4 (1.4–7.5)	2.8 (1.1–4.5)
24 0/7–27 6/7	46.7 (21.3–73.4)	30.0 (12.6–47.4)
28 0/7–29 6/7	0	0
30 0/7–31 6/7	0	0
32 0/7–33 6/7	0.9 (0–5.1)	0.5 (0–2.6)
**Discharge without severe morbidity**	78.3 (72.3–84.4)	84.2 (80.4–88.0)
24 0/7–27 6/7	26.7 (7.8–55.1)	26.7 (9.9–43.5)
28 0/7–29 6/7	47.8 (25.7–69.9)	67.4 (53.3–81.5)
30 0/7–31 6/7	71.4 (55.7–87.2)	80.0 (70.4–89.6)
32 0/7–33 6/7	94.4 (90.0–98.8)	97.2 (95.0–99.4)
**Discharge without moderate-severe morbidity**	35.0 (28.0–42.0)	49.2 (44.0–54.4)
24 0/7–27 6/7	0	3.3 (1.0–17.2)
28 0/7–29 6/7	0	2.2 (1.0–11.5)
30 0/7–31 6/7	20.0 (6.1–33.9)	37.1 (25.5–48.7)
32 0/7–33 6/7	52.3 (42.7–62.0)	69.6 (63.4–75.8)

Data are % (95% CI).

A majority of (91.7%, 165/180) PPROM occurred in the lower sac. When neonatal outcomes are stratified by membrane status, there was no significant difference was found in general neonatal outcomes between PPROM and non PPROM twins (Table 4). PPROM twins had lower rates of respiratory failure (*P* = 0.026), need for mechanical ventilation (*P* = 0.015) and admission to NICU (*P* = 0.021) compared to non PPROM twins.

**Table 4:** Neonatal outcomes of twin pregnancies with PPROM by membrane status.

Variable	PPROM (*N* = 180)	Non-PPROM (*N* = 180)	*P*
Patent ductus arteriosus	69 (38.5)	77 (43.0)	0.390
Intraventricular hemorrhage	13 (7.3)	15 (8.4)	0.694
Bronchopulmonary dysplasia	14 (7.8)	14 (7.8)	1.00
Necrotising enterocolitis	25 (14.0)	16 (8.9)	0.135
Sepsis	41 (22.9)	39 (21.8)	0.800
Respiratory failure	52 (29.1)	72 (40.2)	0.026
Severe pneumonia	12 (6.7)	17 (9.5)	0.333
Respiratory distress syndrome	54 (30.2)	66 (36.9)	0.179
Need for mechanical ventilation	81 (45.3)	104 (58.1)	0.015
Neonatal or foetal death	6 (3.3)	4 (2.2)	0.521
Discharge without severe morbidity	148 (82.2)	155 (86.1)	0.312
Discharge without moderate-severe morbidity	95 (52.8)	82 (45.6)	0.171
5-min Apgar score < 7	5 (2.8)	7 (3.9)	0.557
Admission to NICU	44 (24.6)	64 (35.8)	0.021
Neonatal hospital stay (d)	17 (10–27)	17 (10–30)	0.798
Birth weight (Kg)	1.74 (1.48–1.91)	1.77 (1.48–1.97)	0.579
Neonatal hospitalisation Cost (thousand CNY)	20 (11–34)	20 (12–35)	0.567

Data are median (Q1-Q3) or N (%).

*GA* gestational age, *NICU* neonatal intensive care unit, *CNY* China Yuan.

### Logistic regression analysis

Results of the multivariate analysis of discharge without severe or moderate-severe morbidity are presented in Table 5. A total of 303 (84.2%) neonates survived to hospital discharge without severe morbidity and 177 (49.2%) without moderate-severe morbidity. Binary logistic regression analysis indicated that GA at PPROM (OR, 1.16; 95% CI, 1.12–1.20 for each day) and latency period (OR, 1.16; 95% CI, 1.09–1.24 for each day) were positively associated with survival to hospital discharge without moderate-severe neonatal morbidity. GA at PPROM (OR, 1.12; 95% CI, 1.09–1.16), latency period (OR, 1.11; 95% CI, 1.01–1.23), 5-min Apgar score > 7(OR, 8.22; 95% CI, 1.27–53.43), and absence of any pregnancy complications (OR, 2.93; 95% CI, 1.28–6.71) were positively associated with survival to hospital discharge without severe neonatal morbidity. Chorionicity, clinical chorioamnionitis, and complete steroid treatment were not associated with neonatal morbidity and mortality.

**Table 5:** Binary logistic regression analysis of discharge without severe and moderate-severe morbidity.

Variables	Comparison	Discharge without moderate-severe morbidity		Discharge without severe morbidity	
		OR (95% CI)	*P* value	OR (95% CI)	*P* value
GA at PPROM(d)	Continuous	1.16 (1.12–1.20)	< 0.01	1.11 (1.09–1.16)	< 0.01
Latency period (d)	Continuous	1.16 (1.09–1.24)	< 0.01	1.11 (1.01–1.23)	0.035
Complete steroids treatment	Yes reference: no	1.23 (0.72–2.25)	0.412	2.42 (0.97–6.03)	0.059
Chorioamnionitis	Yes ref: no	0.79 (0.29–2.13)	0.637	1.35 (0.50–3.68)	0.554
Chorionicity	DC ref: MC	0.61 (0.29–1.28)	0.192	0.98 (0.29–3.36)	0.910
5-min Apgar score	≤ 7 ref: > 7	8.60 (0.84–88.13)	0.070	8.22 (1.27–53.43)	0.027
Pregnancy complications	Presence ref: absence	0.93 (0.54–1.59)	0.787	2.93 (1.28–6.71)	0.011

OR > 1 indicates improved odds of discharge without severe and moderate-severe morbidity relative to the comparison group.

*OR* odds ratio, *GA* gestational age, *PPROM* pretem premature rupture of membranes, *DC* dichorionic, *MC* monochorionic.

## Discussion

The present study describes the maternal and neonatal characteristics of twin pregnancies complicated by PPROM. When twin pregnancies experienced PPROM at 24 0/7 to 33 6/7 weeks’ gestation, half of the cases delivered within 24 h after PPROM and one in 10 cases remained undelivered for 7 days or more. Obstetric and neonatal outcomes were reported by GA at PPROM; as GA at PPROM increased, the risk of adverse neonatal outcomes decreased, especially after 32 weeks. We concluded that GA at PPROM and latency period were positively related to hospital discharge without severe or moderate-severe neonatal morbidity, which is consistent with the findings of previously published studies^11,12^. These results can help provide individualised consultation at the moment of admission.

Several previous studies have elucidated the issue of PPROM in twin pregnancies, but the majority focused on comparing the latency period, clinical characteristics, and outcomes between twin and singleton pregnancies^6,7,9^. These studies reported that PPROM in twin pregnancies has a shorter latency period than singleton pregnancies, which suggests that the pathogenesis and therapeutic regimen may differ between twin and singleton pregnancies. In this study, the median latency period in twins with PPROM was 20 h, which was shorter than that reported in other studies^6,7,11^. The main reason for this is that the median GA at PPROM was 32 2/7 weeks in this cohort, which was negatively correlated with the latency period; however, the mean GA at PPROM was 29.1 weeks and at delivery was 29.3 weeks in previous studies^6,7^. Furthermore, in a study of 49 twin pregnancies, the median GA at PPROM was 31 weeks and the median latency period was 0 days, which are similar to our observations^10^.

A earlier GA at PPROM increased the risk of chorioamnionitis and cord prolapse; this result was attributed to the prolonged latency that allowed microorganisms more time to enter the uterine cavity and umbilical cord more time to prolapse after PPROM^13^. Twin pregnancies are less likely than singleton pregnancies to develop clinical chorioamnionitis or placental abruption following PPROM^7^. The data of obstetric outcomes containing clinical chorioamnionitis, placental abruption, and cord prolapse in this cohort were slightly difference from those of other reports in twin pregnancies with PPROM^6,7,14^. In this cohort, 91% of pregnant women delivered by caesarean section, a rate that was significantly higher than the 60% reported by Mendez-Figueroa et al^15^. The reason for this discordant result may be that three-quarters of twins are conceived through assisted reproductive technology, and their expectant mothers are concerned of adverse neonatal outcomes during vaginal delivery and tend to choose caesarean section.

In this study, no newborn was diagnosed with periventricular leukomalacia during hospitalisation. Yu et al. investigated the cases of PPROM occurring before 34 weeks’ gestation and noted periventricular leukomalacia in 1.1%^11^. In a multi-centre randomised trial of women with twin pregnancies and PPROM between 24 0/7 and 31 6/7 weeks’ gestation, 2.4% had periventricular leukomalacia^15^. Regarding other major neonatal morbidities mentioned in this article, our data are somewhat different from those of the other two studies on PPROM^11,15^. The difference in the inclusion and exclusion criteria, primary endpoints, and therapeutic schedule makes comparisons among the studies challenging.

There was no significant difference between PPROM and non PPROM twins in general neonatal outcomes, Cohen et al. had similar conclusions^16^. However, we found that foetuses with ruptured sac had lower rates of respiratory failure, need for mechanical ventilation and admission to NICU compared to those with intact membrane sac, this result may be attributed to PROM itself serves as a stressful stimulus to accelerate fetal glucocorticoid production, which in turn induces surfactant production in fetal lungs^17^. A retrospective cohort of 23 multiple pregnancies complicated by PPROM occurring before 26 weeks reported that neonates with PPROM were more likely to experience intrauterine demise^18^. The reason for this discordant result may be that GA at PPROM affected respiratory morbidity. Assessment and comparison of fetal lung maturity in twin pregnancies with PPROM is our next research direction.

Upon controlling for other confounding factors, we identified two variables—GA at PPROM and latency period—were independently correlated with neonatal mortality or morbidity. GA is recognised as an important factor affecting neonatal outcomes^11,12,19^, our results indicated that an earlier GA at PPROM was associated with worse perinatal outcomes, whether the GA was considered a continuous variable or divided into < 32 weeks versus ≥ 32 weeks. Similarly, Nayot et al. reported that PPROM at < 32 weeks’ gestation with short latency was associated with increased incidence of severe neonatal morbidity in singleton pregnancies^12^. The rate of neonatal survival to hospital discharge was 97.2%, and 84.2% who were discharged without severe neonatal morbidity in the present study included twin pregnancies with PPROM occurring at 24 0/7 to 33 6/7 weeks’ gestation. However, the corresponding values in twins with PPROM before 26 0/7 weeks were only 43% and 17%^18^. There are two statements on latency period: one viewpoint states that a prolonged latency period decreases the incidence of neonatal mortality or morbidity^11,12^, while the other deems that neonatal outcomes were not affected by latency period after PPROM^20^. Therefore, in the absence of infection or other indications for pregnancy termination, obstetricians should try to extend the GA as far as possible to improve perinatal outcomes and remain alert to the occurrence of chorioamnionitis, cord prolapse, and placental abruption.

Multivariate analysis revealed that the presence of pregnancy complications and a 5-min Apgar score < 7 were independent risk factors for discharge with severe neonatal morbidity. Pregnancy complications are related to each other, and several studies stated that ICP is associated with an increased risk of preeclampsia and gestational diabetes mellitus^21–23^. The presence of gestational diabetes also increases the risk of HDP^24^. However, the impact of gestational diabetes on twin pregnancies outcomes was attenuated compared with that of singleton pregnancies^25^. The neonates of women with gestational hypertension were at significant risk for morbidity, especially ROP and respiratory disorders^26,27^. Women with ICP were at higher risk of preterm birth, meconium-stained amniotic fluid, NICU admission, and stillbirth^28^. Taken together, the above factors lead to an increased risk of neonatal morbidity. Consistent with our conclusion, two large sample studies also revealed that low 5-min Apgar scores are associated with high neonatal morbidity and poor prognosis^29,30^.

The present study is limited by its retrospective design. Also, the long-term outcomes of these neonates are currently unknown, although we plan to explore this next. Secondly, only 55% patients received complete antenatal steroids treatment because of short latency period. Moreover, histological chorioamnionitis was not included in this study due to incomplete data on placental pathology. In addition, we excluded those women who terminated the pregnancy and declined neonatal resuscitation, which are biases that would make our results appear more favourable because these excluded pregnant women would tend to have earlier GA at PPROM.

This study described in detail the obstetric and neonatal outcomes of a large cohort of twin pregnancies with PPROM at 24 0/7 to 33 6/7 weeks’ gestation stratified by GA at membrane rupture and membrane status, which was rarely reported in previous studies. Overall, our results are relatively satisfactory because 84.2% of the neonates survived to hospital discharge without severe morbidity. As GA at PPROM increased, the risk of adverse obstetric and neonatal outcomes decreased, especially after 32 weeks. GA at PPROM and latency period were significantly associated with severe and moderate-severe neonatal morbidity; furthermore, pregnancy complications and a 5-min Apgar score < 7 independently affect severe neonatal morbidity. Thus, we must still identify and intervene with the modifiable factors that could improve perinatal outcomes. Meanwhile, our findings can be used to counsel pregnant women and encourage intrauterine transfer.

## Methods

### Subject selection

This retrospective study included a cohort of twin pregnancies complicated by PPROM between 24 0/7 and 33 6/7 weeks’ gestation admitted to the Obstetrics Department at Chongqing Health Center for Women and Children between January 2017 and April 2021. Pregnancies with the following conditions were excluded: at least one intrauterine death on admission, genetic or structural foetal anomalies, complications of monochorionic diamniotic twins (such as twin-to-twin transfusion syndrome [TTTS], twin reversed arterial perfusion sequence [TRAPS]), or termination of pregnancy. Patients who declined neonatal resuscitation were also excluded. The study was approved by the Ethics Committee of Chongqing Health Center for Women and Children and waived informed consent for retrospective study. We confirmed that all methods were performed in accordance with the relevant guidelines and regulations.

### Diagnostic criteria and definitions

#### PPROM diagnostic criteria

Membrane rupture occurred within 37 weeks’ gestation before the onset of labour. The diagnosis was based on the history of vaginal discharge, and leakage of amniotic fluid from the cervical os or pooling of amniotic fluid in the posterior vaginal vault was observed during on a sterile speculum examination. We performed an ultrasound examination and insulin-like growth factor binding protein-1 or fern tests to assist with the diagnosis PPROM when necessary.

#### Determination of GA, latency period and assessment of chorionicity

GA was determined by the date of the last menstrual period and verified by the first trimester ultrasound; it was estimated by the date of embryo transfer when the pregnancy was conceived through assisted reproductive technology. The identification of chorionicity was assessed by ultrasonographic findings in the first trimester.

The latency period was defined as the time from membrane rupture to delivery in hours or days.

#### Definition of clinical chorioamnionitis

Maternal body temperature ≥ 38℃ accompanied by any of the following criteria meet the diagnostic criteria for clinical chorioamnionitis: malodorous vaginal discharge; foetal tachycardia or maternal tachycardia; uterine tenderness; uterine irritation; or maternal peripheral blood leukocyte count ≥ 15 × 10^9^/L.

### Management of PPROM

All patients of expectant management received a single course of dexamethasone for maturation of the foetal lungs and intravenous broad-spectrum prophylaxis (ampicillin, ceftezole, or clarithromycin) for 48 h, followed by oral amoxicillin and erythromycin for 5 days to prevent infection. Magnesium sulphate was routinely administered for its foetal neuroprotective effects. Tocolytic agents such as nifedipine, indomethacin, atosiban, and ritodrine were administered depending on the status of pregnancy. Complete steroid treatment was defined as four injections of 6 mg dexamethasone administered to the mother at 12-h intervals.

The status of mother and foetus was closely monitored until delivery, with the measurement of vital signs (every 6 h), examination of uterine tenderness daily, and determination of serum inflammatory markers (blood routine examination, C-reactive protein, procalcitonin) (every 3 days). Ultrasound scans were performed every 3–4 days to evaluate the amniotic fluid volume and foetal status. Non-stress tests were started at 32 weeks and earlier for high-risk patients and performed daily. .

When clinical chorioamnionitis was diagnosed, premature birth was inevitable, there were indications for delivery for the mother and/or foetus, or GA reached 34 weeks, the pregnancy was terminated by caesarean section or vaginal delivery depending on the patient’s preference, obstetric history, and foetal situation.

### Data collection

We obtained maternal and foetal information through the patients’ electronic medical records from the hospital information system. The database contains the following parameters: (1) Maternal parameters, including age, working condition, parity, and pre-pregnancy body mass index (kg/m^2^); (2) Obstetric information, including mode of conception, chorionicity, GA at PPROM, complete steroid treatment, use of tocolytic agent, and pregnancy complications (gestational diabetes, intrahepatic cholestasis of pregnancy [ICP], hypertensive disorder of pregnancy [HDP]); clinical chorioamnionitis, placental abruption, cord prolapse, GA at delivery, mode of delivery, and latency period in hours and days; (3) Foetal parameters, including sex, birth weight, 5-min Apgar score, prenatal death, neonatal death, need for mechanical ventilation, admission to the neonatal intensive care unit (NICU), days spent in the hospital, hospitalisation cost, and major neonatal complications (containing patent ductus arteriosus [PDA], IVH, RDS, periventricular leukomalacia, sepsis, NEC, BPD, ROP, respiratory failure, or severe pneumonia). Severe neonatal morbidity was defined as any of the following outcomes: stage IIB or III NEC, grade III or IV IVH, BPD, grade 3 or 4 ROP, periventricular leukomalacia, prenatal or neonatal death. Moderate-severe morbidity was defined as one or more of the following outcomes: IVH, RDS, NEC, BPD, ROP, sepsis, respiratory failure, severe pneumonia, periventricular leukomalacia, and prenatal or neonatal death. We analysed and report the perinatal outcomes for the entire study population as well as by GA at PPROM and membrane status.

To identify factors associated with hospital discharge without severe or moderate-severe morbidity, seven variables (GA at PPROM in days, latency period in days, clinical chorioamnionitis, chorionicity, complete steroid treatment, 5-min Apgar score, any pregnancy complication) were included in the logistic regression analysis.

### Statistical analyses

All data were analysed using SPSS version 25.0 (IBM, Armonk, NY, USA). Descriptive statistics were used to analyse all variables, the chi-squared test or Fisher’s exact test was used to analyse categorical variables. All continuous variables were non-normally distributed, compared with Mann–Whitney U test and summarised as median with interquartile range (IQR). A correlation analysis was used to evaluate the association between GA at PPROM and perinatal outcomes and represented by Kendall’s tau-b correlation coefficient with two-tailed tests. Binary logistic regression analysis was performed to identify independent variables associated with hospital discharge without severe or moderate-severe morbidity. Odds ratios (OR) and 95% confidence intervals (CI) were calculated. Values of *P* ≤ 0.050 were considered statistically significant.

## Data Availability

Data are available upon reasonable request. All data relevant to the study are included in the article.
